# Current Concepts and Investigations of Fracture-Related Infection

**DOI:** 10.1155/2025/9768347

**Published:** 2025-01-28

**Authors:** Nan Jiang, Chang-peng Xu, Paul Stoodley, Martin A. McNally

**Affiliations:** ^1^Division of Orthopaedics & Traumatology, Department of Orthopaedics, Nanfang Hospital, Southern Medical University, Guangzhou 510515, China; ^2^Department of Orthopaedics, Guangdong Second Provincial General Hospital, Guangzhou 510317, China; ^3^Departments of Microbial Infection and Immunity & Orthopaedics, The Ohio State University Wexner Medical Center, Columbus, OH 43210, USA; ^4^National Centre for Advanced Tribology at Southampton (nCATS) and National Biofilm Innovation Centre (NBIC), Department of Mechanical Engineering, University of Southampton, Southampton SO17 1BJ, UK; ^5^Bone Infection Unit, Nuffield Orthopaedic Centre, Oxford University Hospitals, Oxford, UK

Fracture-related infection (FRI) is defined as the infection of the osseous tissue contacting the implant with or without infection of the surrounding soft tissue following implantation of the fracture device, resulting from contamination of pathogens with or without compromised immunity of the host [1]. It is estimated that the average incidence of this disease is approximately 5%, with 1-2% and over 30% following closed and open fractures, respectively [2]. Currently, FRI remains to be one of the most challenging and catastrophic disorders for both clinicians and patients, not only for its long disease course, a high rate of infection recurrence, with a great risk of disability, both physically and psychologically [3], but also for its heavy economic burden [4], both personally and socially.

This Special Issue aimed to solicit original research articles as well as review articles covering current concepts and investigations regarding the fundamental science, clinical characteristics, diagnosis, and treatment of FRI. After in-house and peer-review process, four papers were accepted for publication, including “Uncoated vs. Antibiotic-Coated Tibia Nail in Open Diaphyseal Tibial Fracture (42 according to AO Classification): A Single Center Experience” by T. Greco et al. [5], “The Role of Negative-Pressure Wound Therapy in Patients with Fracture-Related Infection: A Systematic Review and Critical Appraisal” by S. Haidari et al. [6], “Novel Elongator Protein 2 Inhibitors Mitigating Tumor Necrosis Factor-*α* Induced Osteogenic Differentiation Inhibition” by W.-J. Wu et al. [7], and “A bibliometric analysis of clinical research on fracture-related infection” by C. Li et al. [8]

In a retrospective comparative study, Greco et al. [5] evaluated the prophylactic effect of antibiotic coated nail against infection following open diaphyseal tibial fracture (AO type classification: 42). Based on the comparison outcomes of 23 patients using a standard uncoated nail and 23 patients by a gentamicin-coated nail, the authors found that no deep wound infections and good fracture healing in the use of antibiotic-coated nails. Thus, they concluded that antibiotic nails have been shown to play a role in the treatment of fractures in critically ill patients with severe soft tissue damage. This study once again confirmed the definite effect of local antibiotic use in the prevention of osteoarticular infection. However, considering the limited sample size, future multicenter studies with a larger sample size are warranted.

In a systematic review, Haidari et al. [6] summarized the influence of negative-pressure wound therapy (NPWT) on the treatment outcomes of FRI, especially regarding infection recurrence. A total of 12 studies were recruited for analysis, and the results showed that the infection recurrence rate ranged from 2.8% to 34.9%. In addition, the wound healing time varied from 2 to 7 weeks. Finally, the authors indicated that conclusions of the synthesis analyses should be interpreted with caution as the lack of uniformity of the included studies. More important, no clear scientific evidence is existed to support the use of NPWT as a definitive treatment of FRI. The authors only recommended early soft tissue coverage with a local or free flap as soon as possible. Also, NPWT can only be acted as a temporary strategy, which may be safe for a few days as soft tissue coverage until definitive soft tissue management could be performed.

In a fundamental research, Wu et al. [7] used an in silico virtual screening method to select molecules from a chemical drug molecule library that bind to Elongation protein 2 (ELP2), a novel drug target in the inflammatory microenvironment generated by TNF-*α* induction, and altogether 95 candidates were obtained preliminarily. Further analysis showed that two molecular compounds (candidates 2 # and 5 #), which could bind to ELP2, were able to rescue osteoblast differentiation in the inflammatory microenvironment by TNF-*α*. Therefore, they concluded that the findings of candidates 2 # and 5 # may facilitate further optimization and development for potential clinical treatment of inflammation-mediated orthopaedic diseases.

In a bibliometric analysis, Li et al. [8] described the trends in clinical research related to FRI published between the year 2000 and 2020. A total of 2597 records from 89 countries were included for analysis. Outcomes revealed that authors from the United States of America (USA) published the highest number of articles and citations. International collaborations were present among 72 countries, with the most active country being the USA. The most contributive institution was the University of California. The highest number of papers and citations was from the Injury and the Journal of Orthopaedic Trauma. The top 100 most cited articles were published in 27 different journals, with the number of citations ranging between 97 and 1004. The latest trend topics were related to FRI diagnosis. This study displayed the research characteristics and trends of FRI from multiple perspectives. Finally, the authors stated that as growing number of investigations focusing on FRI, consensus among scientists and clinicians that standardization regarding this topic is essential.

As a disorder of high heterogeneity, pathogenesis of FRI is complex, which associate with both intrinsic and extrinsic factors. More studies should be conducted to investigate potential factors involving in the development FRI. Meanwhile, clinical investigations with a high level of evidence should be performed to optimize diagnosis, treatment, and prognosis of this disorder.

*Nan Jiang* *Chang-peng Xu* *Paul Stoodley* *Martin A. McNally*
